# Supplementary material for: Faf2 is required for neural differentiation in embryonic neural progenitor cells
Source: bioRxiv. 2026 Jul 13:2026.07.12.737973. Preprint. [Version 2] doi: 10.64898/2026.07.12.737973 (PMC13404890; doi:10.64898/2026.07.12.737973)
Supplement: Supplementary file 1 [file NIHPP2026.07.12.737973v2-supplement-5.pdf]

bioRxiv preprint doi: https://doi.org/10.64898/2026.07.12.737973; this version posted July 13, 2026. The copyright holder for this preprint (which was not certified by peer review) is the author/funder, who has granted bioRxiv a license to display the preprint in perpetuity. It is made available under a CC-BY 4.0 International license.

Supplementary Figure 1. Faf2 Knockout generation

A) Gene map of *Faf2* shows exon 4 deletion target (red) between two red arrowheads. Red arrowheads indicate sgRNA target cut sites. Black arrowheads indicate primer positions for genotyping. Sequence from Sanger sequencing upstream and downstream of sgRNA sequences are shown for WT cells and in KO deletion cells. The sequencing trace is shown for the deletion product.
B) 1.5% agarose gel showing genotyping results of WT and *Faf2 KO* genomic DNA. A product at 1235bp indicates WT allele and 520bp indicates deletion allele.
C) Protein and domain map of wildtype FAF2 (top). Predicted protein structure for the *Faf2 KO* cells from DNA sequence in which the protein sequence is altered at the position of the red arrowhead, followed by nonsense mutation (bottom).
D) Representative western blot of unfolded protein response proteins (p-IRE1 110kDa, BIP 75 kDa, ATF6 50kDa, p-eIF2a 38kDa, GAPDH 37kDa) in WT and KO cells.

Supplementary Figure 2. Gene ontology results from genes that call transcription factors.

bioRxiv preprint doi: https://doi.org/10.64898/2026.07.12.737973; this version posted July 13, 2026. The copyright holder for this preprint (which was not certified by peer review) is the author/funder, who has granted bioRxiv a license to display the preprint in perpetuity. It is made available under a CC-BY 4.0 International license.

Genes that called transcription factors in Figure 2 were input to gProfileR for gene ontology analysis. GO: Biological Process terms are reported here for each transcription factor.

Supplementary Figure 3. Knocking down Faf2 inhibits neural differentiation

A) Model of shRNA plasmids and strategy for detecting random genomic integration by PCR. Black arrows indicate primer locations for genotyping.
B) Genotyping PCR for polyclonal shFAF2 line. Band at 230bp indicates presence of shRNA plasmid. Band at ~300bp is an artifact band found in WT cells. Primer sets both used common forward primer on U6 promoter and shRNA target-specific reverse primers to identify the two shRNAs that were incorporated.
C) Protein and domain map of where each shRNA targets FAF2.
D) Western blot against FAF2 and GAPDH in WT and *shFaf2* cells.
E) Immunofluorescence against FAF2 in WT and *shFaf2* cells.
F) Western blot against BIP and GAPDH in WT and *shFaf2* cells.
G) Immunofluorescence against FAF2 (magenta) and TUBB3 (green). Cells were induced to differentiate with RA.
H) Quantification of pixel intensity from images as in (G)
I) Immunofluorescence against FAF2 (magenta) and TUBB3 (green) in WT, untreated *shFaf2*, and 4-PBA treated *shFaf2* cells. Cells were induced to differentiate with RA.
J) Quantification of TUBB3 pixel intensity.

SUPPLEMENTARY FIGURE 1

bioRxiv preprint doi: https://doi.org/10.64898/2026.07.12.737973; this version posted July 13, 2026. The copyright holder for this preprint (which was not certified by peer review) is the author/funder, who has granted bioRxiv a license to display the preprint in perpetuity. It is made available under a CC-BY 4.0 International license.

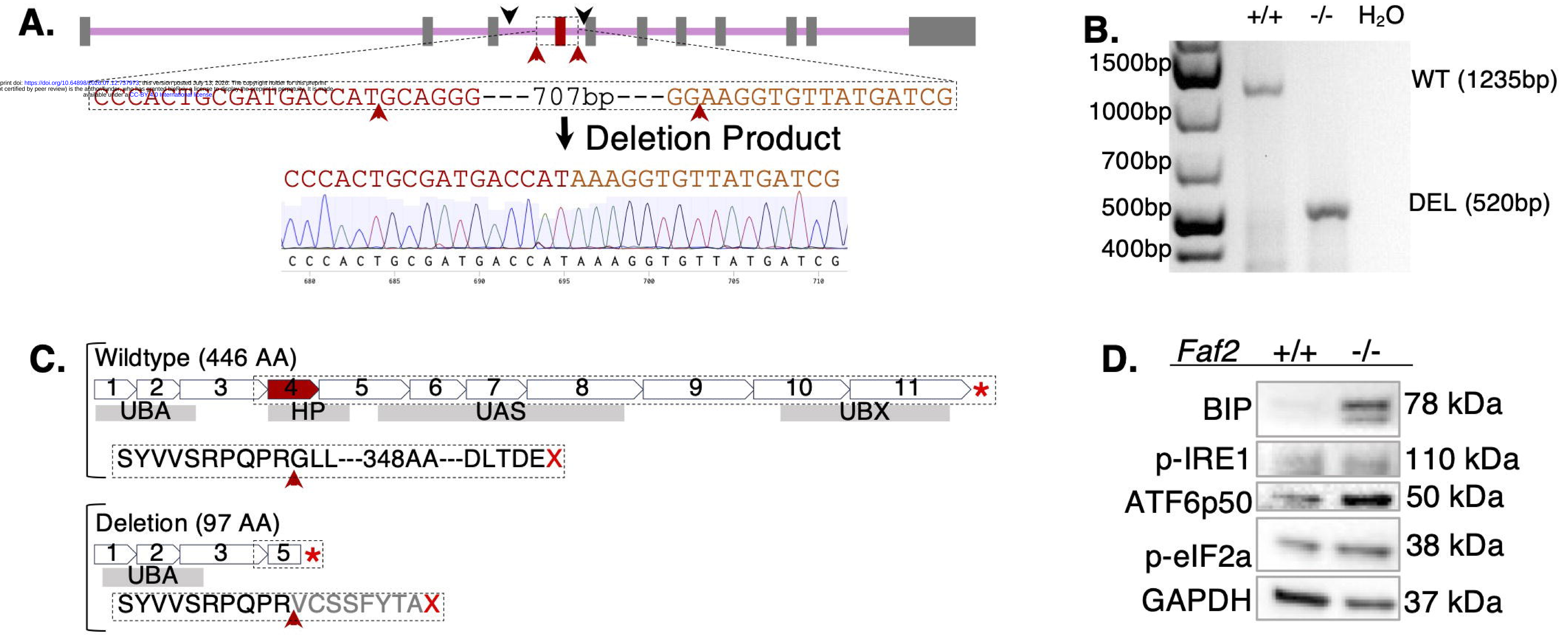

SUPPLEMENTARY FIGURE 2

Highlighted GO terms from gProfiler

XBP-1 DOWN

id	source	term_id	term_name	term_size	intersection_size	p_value
1	GO:BP	GO:0048522	positive regulation of cellular process	5332	178	5.7e-13
2	GO:BP	GO:0048518	positive regulation of biological process	5712	184	2.4e-12
3	GO:BP	GO:0048856	anatomical structure development	5738	174	4.1e-09
4	GO:BP	GO:0009987	cellular process	20290	434	4.1e-09
5	GO:BP	GO:0032502	developmental process	6327	186	5.0e-09
6	GO:BP	GO:0071840	cellular component organization or biogenesis	7264	203	2.7e-08
7	GO:BP	GO:0048731	system development	3806	126	3.0e-08
8	GO:BP	GO:0007275	multicellular organism development	4535	143	3.0e-08
9	GO:BP	GO:0006996	organelle organization	3041	107	3.9e-08
10	GO:BP	GO:0051179	localization	4803	148	5.0e-08
11	GO:BP	GO:0051128	regulation of cellular component organization	2251	86	6.9e-08
12	GO:BP	GO:0050794	regulation of cellular process	11716	288	6.9e-08
13	GO:BP	GO:0019222	regulation of metabolic process	5875	170	1.2e-07
14	GO:BP	GO:0016043	cellular component organization	7076	195	1.5e-07
15	GO:BP	GO:0050789	regulation of biological process	12137	294	1.6e-07
16	GO:BP	GO:0080090	regulation of primary metabolic process	4471	138	1.7e-07
17	GO:BP	GO:0048519	negative regulation of biological process	5147	153	1.8e-07
18	GO:BP	GO:0009893	positive regulation of metabolic process	3012	103	2.4e-07
19	GO:BP	GO:0065007	biological regulation	12498	299	3.1e-07
20	GO:BP	GO:0048523	negative regulation of cellular process	4924	147	3.1e-07
21	GO:BP	GO:0051130	positive regulation of cellular component organization	1006	49	3.1e-07
22	GO:BP	GO:0007010	cytoskeleton organization	1237	54	1.9e-06
23	GO:BP	GO:0097435	supramolecular fiber organization	590	34	1.9e-06
24	GO:BP	GO:0009653	anatomical structure morphogenesis	2586	89	2.6e-06
25	GO:BP	GO:0007399	nervous system development	2310	82	2.7e-06
26	GO:BP	GO:0030154	cell differentiation	4372	130	4.0e-06
27	GO:BP	GO:0010604	positive regulation of macromolecule metabolic process	2783	93	4.0e-06
28	GO:BP	GO:0048869	cellular developmental process	4373	130	4.0e-06
29	GO:BP	GO:0048583	regulation of response to stimulus	3487	108	1.2e-05
30	GO:BP	GO:0009889	regulation of biosynthetic process	5074	143	1.6e-05
31	GO:BP	GO:0060255	regulation of macromolecule metabolic process	5454	150	3.1e-05
32	GO:BP	GO:0033043	regulation of organelle organization	1028	44	5.8e-05

g:Profiler (biit.cs.ut.ee/gprofiler)

XBP-1 UP

id	source	term_id	term_name	term_size	intersection_size	p_value
1	GO:BP	GO:0051179	localization	4803	177	4.3e-12
2	GO:BP	GO:0009056	catabolic process	2141	99	9.0e-11
3	GO:BP	GO:0006810	transport	3943	149	1.1e-10
4	GO:BP	GO:0051234	establishment of localization	4145	153	2.4e-10
5	GO:BP	GO:0033036	macromolecule localization	2622	111	2.6e-10
6	GO:BP	GO:0048523	negative regulation of cellular process	4924	170	1.4e-09
7	GO:BP	GO:0048518	positive regulation of biological process	5712	188	3.1e-09
8	GO:BP	GO:0080090	regulation of primary metabolic process	4471	157	3.1e-09
9	GO:BP	GO:0048522	positive regulation of cellular process	5332	178	4.0e-09
10	GO:BP	GO:0032502	developmental process	6327	201	7.2e-09
11	GO:BP	GO:0033554	cellular response to stress	1666	78	7.5e-09
12	GO:BP	GO:0048519	negative regulation of biological process	5147	172	7.9e-09
13	GO:BP	GO:0048856	anatomical structure development	5738	186	9.7e-09
14	GO:BP	GO:0019219	regulation of nucleobase-containing compound metabolic process	3450	126	4.4e-08
15	GO:BP	GO:0060255	regulation of macromolecule metabolic process	5454	176	6.1e-08
16	GO:BP	GO:0019222	regulation of metabolic process	5875	186	6.3e-08
17	GO:BP	GO:0006996	organelle organization	3041	114	7.9e-08
18	GO:BP	GO:0051641	cellular localization	2112	88	8.0e-08
19	GO:BP	GO:0008104	intracellular protein localization	2217	91	8.0e-08
20	GO:BP	GO:0051649	establishment of localization in cell	1713	76	9.5e-08
21	GO:BP	GO:0007275	multicellular organism development	4535	152	1.0e-07
22	GO:BP	GO:0006950	response to stress	3596	128	1.0e-07
23	GO:BP	GO:0048869	cellular developmental process	4373	147	1.7e-07
24	GO:BP	GO:0030154	cell differentiation	4372	147	1.7e-07
25	GO:BP	GO:0071840	cellular component organization or biogenesis	7264	215	3.2e-07
26	GO:BP	GO:0016043	cellular component organization	7076	210	4.5e-07
27	GO:BP	GO:0051252	regulation of RNA metabolic process	3196	115	4.6e-07
28	GO:BP	GO:0048583	regulation of response to stimulus	3487	122	6.7e-07
29	GO:BP	GO:0019538	protein metabolic process	3854	131	8.6e-07

g:Profiler (biit.cs.ut.ee/gprofiler)

ATF4 UP

bioRxiv preprint doi: https://doi.org/10.64898/2026.07.12.737973; this version posted July 13, 2026. The copyright holder for this preprint (which was not certified by peer review) is the author/funder, who has granted bioRxiv a license to display the preprint in perpetuity. It is made available under a CC-BY 4.0 International license.

id	source	term_id	term_name	term_size	intersection_size	p_value
1	GO:BP	GO:0033554	cellular response to stress	1666	48	1.6e-06
2	GO:BP	GO:0080090	regulation of primary metabolic process	4471	91	1.6e-06
3	GO:BP	GO:0051179	localization	4803	95	1.7e-06
4	GO:BP	GO:0019222	regulation of metabolic process	5875	108	3.5e-06
5	GO:BP	GO:0048522	positive regulation of cellular process	5332	99	1.1e-05
6	GO:BP	GO:0097190	apoptotic signaling pathway	560	24	1.1e-05
7	GO:BP	GO:0060255	regulation of macromolecule metabolic process	5454	100	1.3e-05
8	GO:BP	GO:0009056	catabolic process	2141	52	2.2e-05
9	GO:BP	GO:0048518	positive regulation of biological process	5712	102	2.8e-05
10	GO:BP	GO:0006810	transport	3943	77	6.6e-05
11	GO:BP	GO:0006950	response to stress	3596	72	6.7e-05
12	GO:BP	GO:0051234	establishment of localization	4145	79	9.9e-05
13	GO:BP	GO:0006974	DNA damage response	827	27	1.5e-04
14	GO:BP	GO:0097193	intrinsic apoptotic signaling pathway	285	15	1.9e-04
15	GO:BP	GO:0043065	positive regulation of apoptotic process	459	19	2.0e-04
16	GO:BP	GO:0046907	intracellular transport	1121	32	2.0e-04
17	GO:BP	GO:0033036	macromolecule localization	2622	56	2.0e-04
18	GO:BP	GO:0065007	biological regulation	12498	177	2.3e-04
19	GO:BP	GO:0061919	process utilizing autophagic mechanism	468	19	2.3e-04
20	GO:BP	GO:0006914	autophagy	468	19	2.3e-04

g:Profiler (biit.cs.ut.ee/gprofiler)

ATF6 UP

id	source	term_id	term_name	term_size	intersection_size	p_value
1	GO:BP	GO:0009056	catabolic process	2141	39	6.5e-09
2	GO:BP	GO:0048523	negative regulation of cellular process	4924	53	5.5e-05
3	GO:BP	GO:0019538	protein metabolic process	3854	45	6.4e-05
4	GO:BP	GO:0006914	autophagy	468	14	7.2e-05
5	GO:BP	GO:0048519	negative regulation of biological process	5147	53	7.2e-05
6	GO:BP	GO:0061919	process utilizing autophagic mechanism	468	14	7.2e-05
7	GO:BP	GO:0033036	macromolecule localization	2622	35	7.2e-05
8	GO:BP	GO:0016236	macroautophagy	272	11	7.3e-05
9	GO:BP	GO:0030163	protein catabolic process	816	18	8.7e-05
10	GO:BP	GO:0035556	intracellular signal transduction	2485	33	1.2e-04
11	GO:BP	GO:0008152	metabolic process	13249	97	1.5e-04
12	GO:BP	GO:0009057	macromolecule catabolic process	1152	21	1.5e-04
13	GO:BP	GO:0051179	localization	4803	49	2.0e-04
14	GO:BP	GO:0009894	regulation of catabolic process	859	17	5.4e-04
15	GO:BP	GO:0051246	regulation of protein metabolic process	1183	20	6.8e-04
16	GO:BP	GO:0019941	modification-dependent protein catabolic process	461	12	8.6e-04
17	GO:BP	GO:0009896	positive regulation of catabolic process	456	12	8.6e-04
18	GO:BP	GO:0006511	ubiquitin-dependent protein catabolic process	455	12	8.6e-04
19	GO:BP	GO:0043632	modification-dependent macromolecule catabolic process	463	12	8.6e-04
20	GO:BP	GO:0048583	regulation of response to stimulus	3487	38	8.6e-04

g:Profiler (biit.cs.ut.ee/gprofiler)

SUPPLEMENTARY FIGURE 3

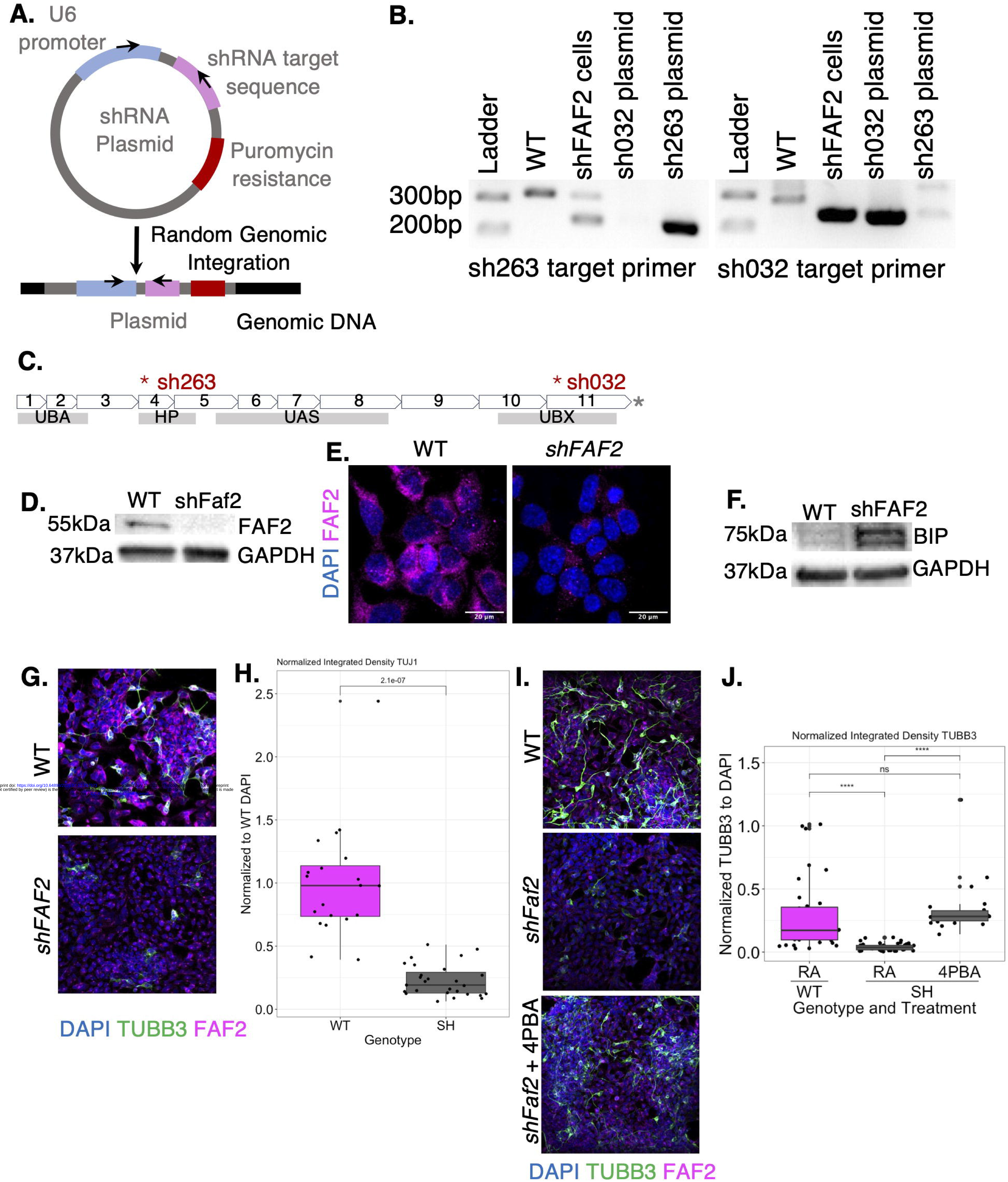

bioRxiv preprint doi: https://doi.org/10.64898/2026.07.12.737973; this version posted July 13, 2026. The copyright holder for this preprint (which was not certified by peer review) is the author/funder, who has granted bioRxiv a license to display the preprint in perpetuity. It is made available under a CC-BY 4.0 International license.
